# WiraChain: a blockchain and FHIR-based platform for improving clinical data interoperability in healthcare

**DOI:** 10.3389/fpubh.2026.1746772

**Published:** 2026-02-10

**Authors:** Leonardo Grau, Joao Urrunaga, José Santisteban

**Affiliations:** 1 Software Engineering, Universidad Peruana de Ciencias Aplicadas, (UPC), Lima, Peru; 2 Information Systems Engineering, Universidad Peruana de Ciencias Aplicadas, (UPC), Lima, Peru

**Keywords:** blockchain, electronic health records, Ethereum, HL7 FHIR, Hyperledger, interoperability, smart contract

## Abstract

Interoperability challenges in healthcare frequently lead to fragmented patient records and duplicated procedures, negatively affecting continuity of care and operational efficiency. Although multiple theoretical frameworks and standards have been proposed to address these issues, practical and fully implemented solutions enabling secure and controlled data exchange remain limited. To address this gap, we developed Wirachain, a decentralized application that integrates blockchain technology with the HL7 FHIR standard for electronic health record management. The system enables patient-controlled permission granting and revocation, incorporates authentication and role-based access control, and supports persistent storage using both FHIR-compliant and traditional databases. Validation was conducted using synthetically generated clinical data and stress-testing scenarios to assess system performance under load. The results demonstrate that the proposed application can reliably manage access permissions and clinical data exchange across interoperable components. The system maintained efficient operation and secure data handling under simulated clinical workflows and increased load conditions. These findings indicate that fully implemented blockchain-based solutions can effectively bridge the gap between conceptual interoperability frameworks and practical healthcare applications. Wirachain illustrates the feasibility of combining blockchain and FHIR standards to support secure, patient-centered, and interoperable clinical operations.

## Introduction

Despite substantial advancements in the digitalization of healthcare, the ongoing lack of interoperability among clinical information systems remains a significant obstacle. Research has shown that improving interoperability can reduce medical errors, enhance institutional resilience, and protect patient data from emerging health threats (1).

This limitation delays access to patient data, leads to redundant testing, and increases the likelihood of clinical errors, thereby compromising the quality and safety of healthcare delivery (2). In Latin America, these challenges are made worse by a continued reliance on informal communication methods, such as paper records, telephone exchanges, and unofficial messaging platforms. This practice, often referred to as user-mediated interoperability (3), fragments the continuity of care and reduces operational efficiency.

To tackle interoperability challenges, various digital frameworks have emerged to facilitate secure and standardized clinical data exchange. One of the most significant standards is HL7 FHIR, which effectively represents medical resources using RESTful APIs and web-based structures. This promotes scalable and flexible communication among different systems (4). Blockchain technology has gained prominence for its ability to ensure traceability and access control through immutable records and authorization mechanisms based on smart contracts (5).

Complementary approaches to securing medical data continue to evolve, such as robust steganography techniques that enhance protection by embedding sensitive information within medical images (6). Furthermore, policy-driven digital health interventions have been systematically reviewed to assess their effectiveness in health promotion and disease prevention, highlighting the importance of aligning technical solutions with broader healthcare policy frameworks to maximize clinical impact (7).

In Peru, electronic health record (EHR) interoperability encounters significant structural, regulatory, and technical challenges. Although there is a legal framework for a national health data registry, its implementation is limited, with interoperability maturity remaining below 10%. Most institutions continue to rely on isolated or paper-based systems, resulting in duplicated tests, delayed diagnoses, and fragmented care (8). The absence of standardized data exchange further diminishes efficiency and undermines the quality and safety of care.

Lack of real-time access to clinical data hinders decision-making, results in duplicated diagnostic procedures, and increases clinical risks, especially in emergencies. Therefore, interoperability is not just about efficiency; it is a vital aspect of patient safety and the reliability of healthcare systems (9).

A promising approach to addressing this crisis involves adopting decentralized architectures, where blockchain technology enables the management of Electronic Health Records (EHRs) with high levels of integrity and access control. Uddin et al. (10) proposed a solution based on Hyperledger Fabric that utilizes private channels and smart contracts for the secure exchange of medical data, eliminating the need for central authorities. Similarly, Díaz and Kaschel (11) created a dual-channel architecture that separates administrative and clinical operations, thereby enhancing throughput and improving access management. Sreejith and Senthil (12) enhanced semantic interoperability by integrating HL7 FHIR with graph-based data models and blockchain authentication. This combination of international standards and decentralized technologies indicates a comprehensive approach where interoperability goes beyond mere data formats to include secure and distributed governance of clinical information.

Despite recent advancements, most studies on blockchain-enabled interoperability are still limited to conceptual models or theoretical frameworks. Research conducted by Uddin et al. (10), Sonkamble et al. (13), and Nkenyereye et al. (14) primarily focuses on logical designs without validating their practicality in real-world healthcare environments. Even experimental efforts, such as those by Díaz and Kaschel (11), remain restricted to backend prototypes and do not include any user-facing components or field testing.

Among the reviewed literature, only a few initiatives have achieved full implementation and validation. Mauricio et al. (15) stand out for developing an interoperable web platform that integrates HL7 FHIR and blockchain technology, which was tested through simulations involving Peruvian clinicians and patients. Similarly, Kim et al. (16) proposed a functional self-sovereign identity model that combines Personal Data Stores with Decentralized Identifiers. However, their approach focuses more on individual record management rather than inter-institutional interoperability.

This gap underscores the disconnect between conceptual design and the practical implementation of clinical interoperability systems. Therefore, this study aims to bridge that divide by developing a functional web application that operationalizes a blockchain-based interoperable architecture. It will also validate the application's performance in real healthcare environments, focusing on usability, security, and patient empowerment.

The objective of this research is to develop WiraChain, a digital platform designed to facilitate the secure and timely exchange of clinical information among healthcare institutions in Peru. The system aims to improve the quality of care, reduce errors caused by fragmented data, and empower patients by granting them controlled access to their medical history. This initiative addresses a significant gap in the Peruvian healthcare sector, which is characterized by low levels of integration and a high degree of system isolation (15).

WiraChain is a decentralized web application that uses HL7 FHIR for technical interoperability, blockchain for managing patient-controlled data, and a REST API as a standardized interface, addressing two critical challenges in emerging healthcare systems such as those in Peru.

This paper is organized into several sections. First, it introduces the research context and motivation behind the study. Next, it reviews related work and identifies gaps in the existing literature. Following that, the proposed methodology is outlined. The paper then presents and discusses the results. Finally, it concludes by summarizing the main findings and suggesting directions for future research.

## Related works

The following discussion synthesizes a compendium of related and comparable research pertinent to the present study.

### Architectures and frameworks

Recent literature demonstrates growing interest in integrating blockchain technologies with interoperability standards such as HL7 FHIR. Although several studies provide solid conceptual and architectural frameworks, most remain theoretical and lack validation in real clinical environments. For instance, Díaz and Kaschel (11) proposed a dual-channel Hyperledger Fabric architecture evaluated through Caliper, yet their work did not extend to clinical interfaces or FHIR integration. Similarly, Uddin et al. (10) developed a modular Fabric-based framework employing smart contracts, though their research focused primarily on system design rather than empirical testing.

From a security and access control perspective, Yaqub et al. (17) introduced a policy-based access control (PBAC) mechanism implemented with blockchain smart contracts, while Sonkamble et al. (13) presented the PCHDM Framework combining SPAKE, IPFS, and Fabric. Despite their technical rigor, neither of these solutions has undergone clinical or operational validation.

In the context of the Internet of Medical Things (IoMT), conceptual frameworks have been proposed to improve distributed management of Electronic Health Records (EHRs). Nkenyereye et al. (14) examined distributed nodes for EHR handling without providing a technical implementation, whereas Gulzar et al. (18) designed a theoretical model–BlockMed–leveraging artificial intelligence for HL7-FHIR translation, but without a deployable prototype.

Emerging studies have explored privacy and scalability through advanced technologies. Ma and Zhang (19) proposed integrating ZK-Rollup and IPFS for privacy-preserving medical records, while Pokharel et al. (1) investigated blockchain-based cybersecurity frameworks emphasizing compliance with HIPAA and GDPR. Similarly, Benaich et al. (20) expanded this line of research by incorporating post-quantum cryptography, decentralized autonomous organizations (DAOs), and artificial intelligence, though their results remain theoretical. Van Wensel and Seneviratne (21) further contributed by proposing a semantic approach that generates smart contracts from HL7-based Knowledge Graphs through an off-chain pipeline, yet their system has not achieved clinical interoperability.

Comprehensive analyses, such as the survey by Merhej et al. (22), offer systematic overviews of blockchain and AI integration in healthcare, identifying challenges related to scalability, interoperability, and regulatory compliance. In contrast, Carter et al. (23) made a more practical contribution through the “OpenPharma Blockchain on FHIR” framework, which employs Ethereum, FHIR standards, and voice biometrics to enable secure, read-only health data exchange while avoiding the storage of protected information on-chain.

In summary, current research establishes a solid theoretical and architectural foundation for combining blockchain and FHIR in healthcare. However, a notable gap persists: few studies provide fully integrated, functional implementations validated within clinical environments, highlighting the need for practical, interoperable, and empirically tested solutions.

### Applications

Recent research has increasingly focused on integrating blockchain technology with HL7 FHIR to improve interoperability, security, and privacy in Electronic Health Records (EHRs). Bran et al. (24) proposed a framework that utilizes blockchain and IPFS for managing allergy and family history data, ensuring secure and interoperable record sharing. In a similar vein, Mauricio et al. (15) developed an architecture and web platform that facilitates the exchange of EHRs among diverse healthcare systems in Peru, without the need to modify existing legacy infrastructures. Uddin et al. (10) developed an architecture based on Hyperledger Fabric to ensure data integrity and decentralization in the management of medical records. Meanwhile, Sreejith et al. (12) presented an HL7 FHIR framework that combines GraphMap and blockchain-based authentication through smart contracts to enhance interoperability in e-health environments.

Recent studies have expanded efforts toward creating decentralized and semantically rich systems. Kim et al. (16) introduced a decentralized personal health record (PHR) management model that integrates personal data storage (PDS) and decentralized identifiers (DID), enabling users to have complete ownership of their data. Meanwhile, Van Wensel and Seneviratne (21) investigated the automated generation of smart contracts from semantic knowledge graphs, aiming to enhance compliance and alignment with standards such as HL7 FHIR. Likewise, Marfoglia et al. (25) proposed a modular pipeline for transforming clinical data into the FHIR format. This approach enhances semantic interoperability and promotes code reuse. Additionally, Gulzar et al. (18) introduced BlockMed, a framework that integrates blockchain technology with artificial intelligence to facilitate secure interoperability and automate the translation between HL7 and FHIR standards.

As shown in Table 1, Mauricio et al. (15) describe one of the few instances in Peru where an interoperable web application that utilizes HL7 FHIR and IPFS, supported by blockchain technology, has been developed, as detailed in Table 1. The system was validated in a simulated environment involving both doctors and patients, demonstrating promising results in terms of traceability and access control. However, its geographical reach is still limited, and the study does not yet address issues related to multi-node scalability or the potential for replication in real hospital settings.

**Table 1:** Comparative summary of blockchain–FHIR applications for EHR interoperability.

**References**	**Contribution**	**Method**	**Result**	**Challenges**
Uddin et al. (10)	Designs architecture using Hyperledger Fabric for secure, decentralized medical record management, ensuring integrity, privacy, and interoperability	Hyperledger Fabric Blockchain, Private Permissioned Blockchain, Membership Service Provider	Restricted secure network for medical data; integrity and traceability guaranteed; interoperability with legacy HL7/CDA; optimized performance	Verify data integrity through end-to-end consistency checks.
Sreejith and Senthil (12)	Proposes HL7 FHIR framework using GraphMap and smart contract authentication for interoperable e-health systems	MongoDB, FHIR, SmartContracts, GraphQL	Better response time; lower CPU/memory usage; greater scalability	Multiple technology complexity; blockchain computational overhead
Mauricio et al. (15)	Proposes a web architecture and application for exchanging EHRs between heterogeneous systems in Peru, without altering existing systems	HL7 FHIR standardization, IPFS, SmartContracts, Ethereum, Node.js	High adoption and usability; acceptable performance; latency +0.35 ms	Latency increases with transactions; complexity integrating HL7 FHIR
Kim et al. (16)	Proposes decentralized PHR system with personal data store (PDS) and decentralized identifiers (DID) for privacy, security, and user control	Personal Data Store (PDS), Decentralized Identifiers (DID), Dynamic Access Key (AK)	DID/key generation < 1s; read/write/update on 500 MB PHR ≈2s	Missing data integrity verification; limited legacy system interoperability; needs speed optimization
Gulzar et al. (18)	Proposes BlockMed, integrating blockchain and AI for secure interoperability and automated HL7–FHIR translation in EHRs	Systematic literature review, AI-driven mapping, blockchain-based consent management	99.5% precision in data mapping; latency ~150 ms.	Scalability, cross-chain interoperability, performance under load
Van WCnsel and Seneviratne (21)	Method to generate smart contracts from semantic knowledge graphs, enabling declarative logic and interoperability using HL7 FHIR standards	Knowledge Graphs, OWL2 ontologies, Notation3, Graph-based	Generated smart contracts for three Medicare cases	Limited support for complex rules; high public blockchain costs; inefficient multivalued property handling
Bran et al. (24)	Proposes an interoperable and secure framework for EHR using Blockchain, IPFS, and HL7 FHIR, applied to allergy records and family history	Hyperledger Sawtooth, IPFS, HL7 FHIR, Azure, PBFT, JMeter	~8 TPS in writing/reading; Latency 5.926–51.836 ms; >80% of patients and 60% professionals support system interoperability	Latency increases with transactions; writing slower than reading
Marfoglia et al. (25)	Develops modular pipeline for transforming clinical data to FHIR, enhancing semantic interoperability and code reusability	Modular ETL pipeline, FHIR Mapping Language, data refinement/sanitization	Converted 1,962 hospital records to 15 FHIR resource types	Lack of regulation; proprietary formats; non-portable tools

Additionally, after conducting a systematic review of indexed databases such as Scopus, Web of Science, IEEE Xplore, and PubMed, using the search string: “(healthcare interoperability OR clinical data exchange OR EHR interoperability) AND (blockchain OR decentralized architecture OR Hyperledger Fabric OR smart contracts) AND (HL7 FHIR OR Fast Healthcare Interoperability Resources OR FHIR API) AND (web application OR functional implementation OR system prototype OR user validation),” the only relevant study identified was conducted by Mauricio, who developed an electronic health record system utilizing EHRs and blockchain technology. This study presented various architectures, designs, and prototypes (mockups). In contrast, this paper proposes a solution based on the FHIR standard that incorporates a more user-friendly interface. Additionally, it places special emphasis on information security, ensuring that user data can only be viewed and modified by entities authorized by the user.

## WiraChain platform model

**Figure 1:**
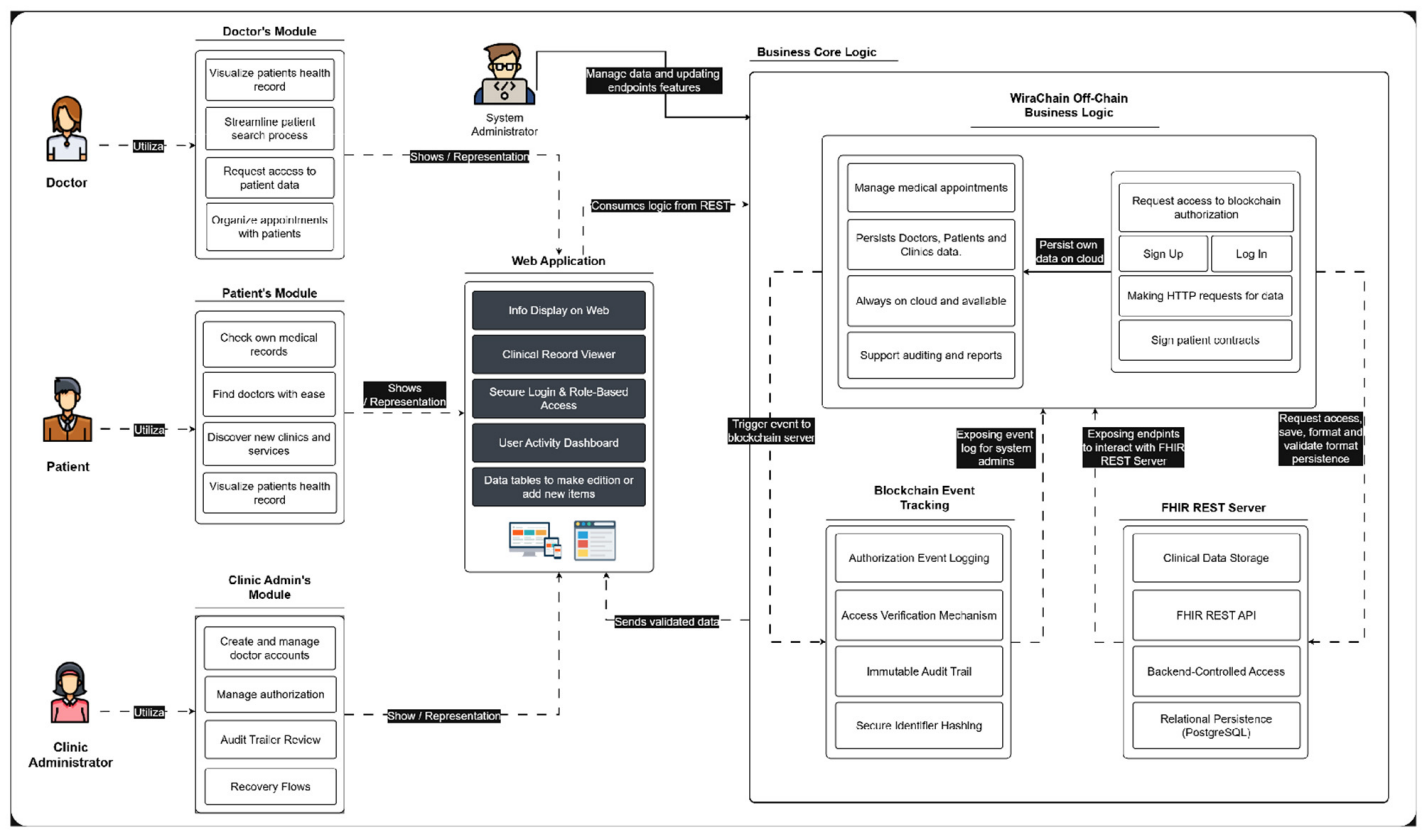
WiraChain model.

This section presents the WiraChain platform model (Figure 1), which is designed to ensure the secure and efficient sharing of clinical data among healthcare institutions in Peru. The model includes four user categories, each with defined roles: (1) patient, who manages personal records and controls data-sharing permissions; (2) doctor, who reviews authorized patient records and documents new encounters; (3) clinic administrator, who oversees staff management and access to institutional data; and (4) system administrator, who maintains the platform's infrastructure and ensures overall operational integrity.

To address limitations observed in previous studies, WiraChain adopts a hybrid persistence architecture in which blockchain technology is used for permission and authentication management while clinical data reside in conventional database systems optimized for complex queries and high throughput. Blockchain's decentralized ledger confers immutability, transparent audit trails, and fine-grained access control enforcement via smart contracts, strengthening identity and access management beyond traditional centralized models in healthcare settings (26). Research has shown that decentralized role-based access mechanisms on blockchain can significantly enhance confidentiality and prevent unauthorized data access compared with central server models (27). Similarly, blockchain-enabled personal health record platforms demonstrate effective patient-controlled consent and auditability, alleviating trust concerns inherent in conventional systems (28). Prior reviews further conclude that hybrid solutions combining blockchain's immutable permission management with off-chain data storage achieve both security and performance goals in healthcare information exchange, balancing resilience against tampering with efficient data retrieval and processing.

## WiraChain Web App

This section details the conceptual architecture and technological development of WiraChain, emphasizing the integration of blockchain components, FHIR standards, and service orchestration. This foundation creates an interoperable and traceable clinical environment that aligns with decentralized governance principles.

### Logical architecture

**Figure 2:**
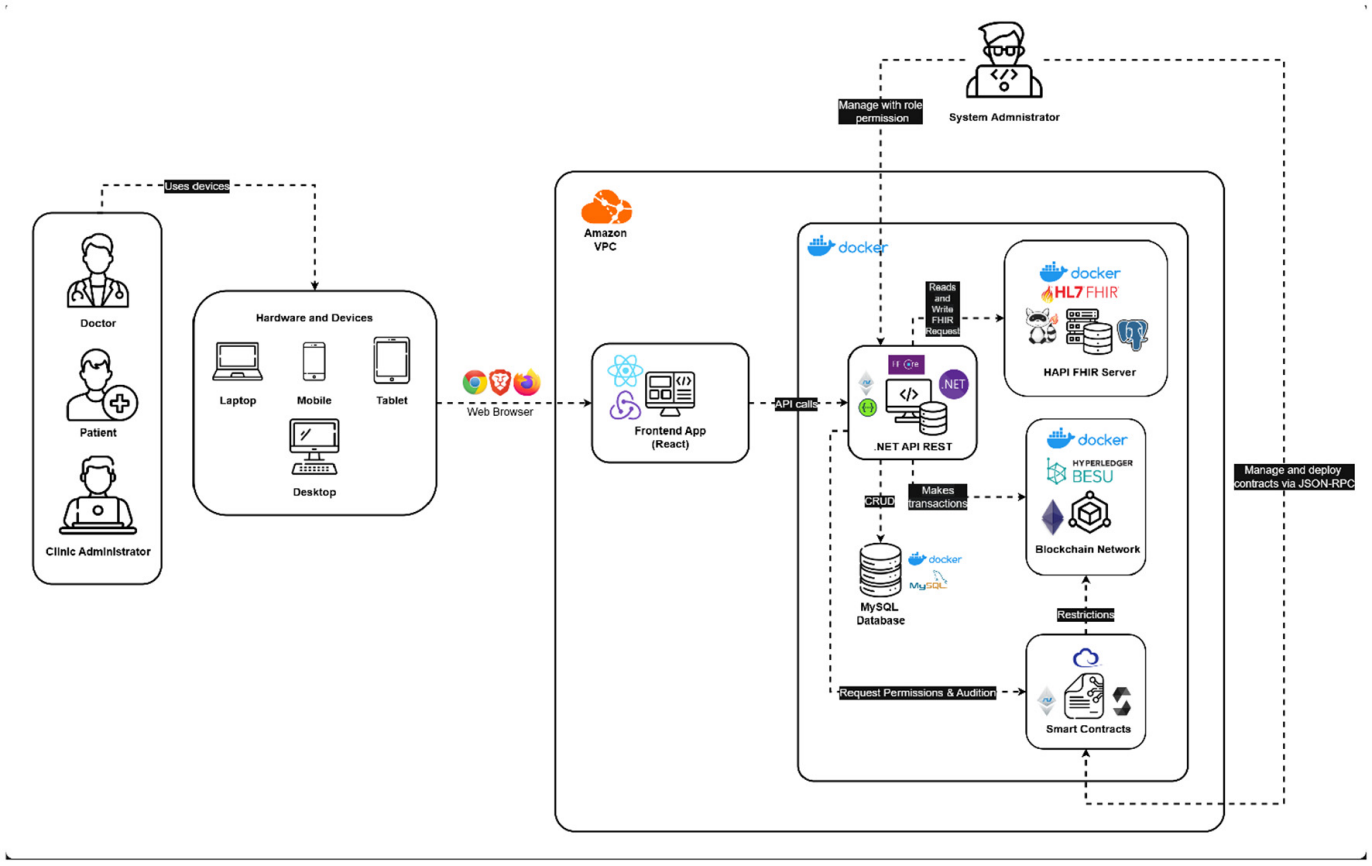
Logical architecture diagram.

WiraChain implements an Interoperability Architecture featuring an Immutable Audit Layer (see Figure 2), designed to ensure the secure and efficient exchange of clinical data. The system integrates React for developing responsive single-page interfaces and the .NET framework for orchestrating operations within a microservice-based environment, enabling robust integration, scalability, and high-performance processing. MySQL provides transactional consistency for identity and credential management, while Hyperledger Besu functions as an immutable audit ledger, ensuring full traceability and regulatory compliance (29).

We adopt Hyperledger Besu, an Ethereum-compatible enterprise blockchain client, as the permissioned ledger for access control and audit logging due to its alignment with the Enterprise Ethereum specification and its native support for fine-grained permissioning under Byzantine fault-tolerant consensus. Unlike other permissioned blockchain platforms that rely on channel-based data isolation and tightly coupled execution models, Besu enables standardized EVM-based smart contracts and seamless integration with service-oriented architectures.

This choice is particularly suitable for WiraChain, where blockchain interactions are intentionally limited to authorization events and immutable audit records rather than high-throughput clinical data processing. In this context, Besu provides a balanced trade-off between audit transparency, cryptographic security, and ecosystem interoperability, while off-chain FHIR services handle time-sensitive clinical workflows and data-intensive operations (30).

The HAPI FHIR framework enables semantic interoperability aligned with the HL7 FHIR standard, ensuring structured clinical data exchange across heterogeneous healthcare systems. FHIR adoption in hybrid architectures has been shown to significantly enhance interoperability and machine-readable data exchange, which is particularly critical in fragmented healthcare environments such as those found in regional clinical networks.

By clearly separating responsibilities—React for interface design, .NET for orchestration, MySQL for transactions, Besu for immutable logging, and FHIR for standardization—the architecture enhances interoperability and data integrity compared with prior monolithic or single-technology stacks. Furthermore, deploying the platform on AWS strengthens security, scalability, and adherence to healthcare data management best practices through managed identity services, encrypted communications, and resilient infrastructure (31).

Notably, backend framework performance benchmarks in peer-reviewed research indicate that compiled frameworks such as ASP.NET Core (C#) demonstrate superior request throughput and lower average response times compared to interpreted Python-based frameworks such as Django and FastAPI in REST API contexts. These performance advantages translate into more efficient request handling, improved scalability under load, and lower server resource utilization—factors that are essential for clinical data systems that must handle high concurrency and low-latency transactions (32).

### Physical architecture

**Figure 3:**
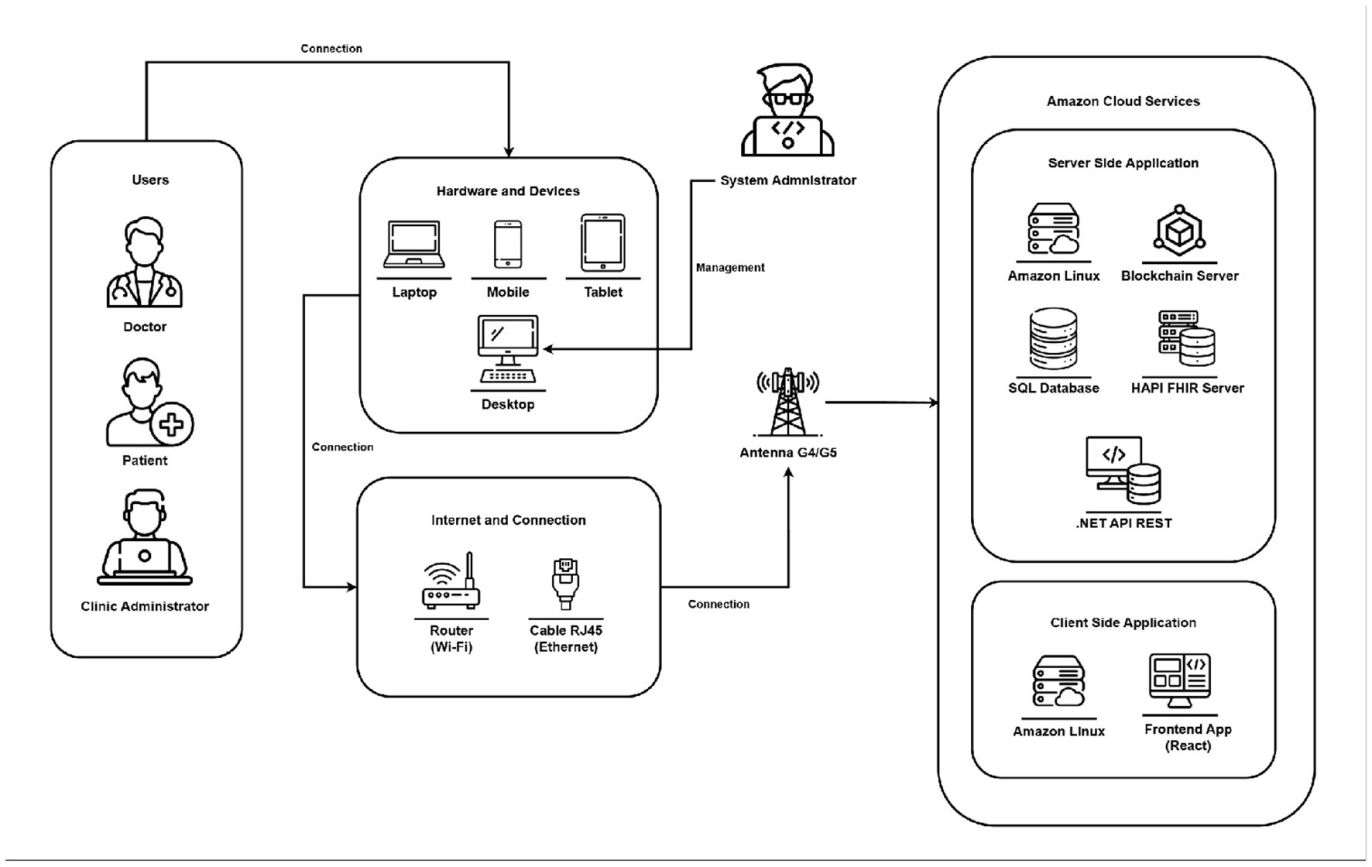
Physical architecture diagram.

Figure 3 illustrates the physical architecture of the system, designed according to modern software engineering principles. The modular and decoupled structure enhances reliability, scalability, and maintainability by preserving data integrity and allowing for the independent evolution of components. This design follows SOLID principles and Infrastructure as Code practices. These features are especially beneficial for decentralized applications, blockchain-based electronic health record (EHR) systems, and FHIR-compatible solutions, where interoperability, security, and traceability are crucial. Overall, the proposed architecture serves as a robust and adaptable foundation for advanced clinical applications (33).

## Development

This section offers a comprehensive overview of the development of the various layers and modules that constitute the system, detailing their structure, functionality, and interaction with one another.

### Off-chain layer

The off-chain layer encompasses all processes executed outside the primary blockchain network, serving as the central hub for business operations and data management across services. Its external execution enhances system performance, reduces network load and transaction costs, and maintains data integrity. In the proposed application, this layer employs a multi-tier architecture comprising logical, visual, and data components: the logical layer governs business rules and blockchain coordination, the visual layer manages user interactions, and the data layer ensures secure off-chain storage. This architecture, grounded in clean architecture and loose coupling principles, promotes modularity, scalability, and maintainability, while the clear division of on-chain and off-chain functions supports efficiency and interoperability (34).

### On-chain layer

The WiraChain blockchain layer underpins the platform's security and auditing framework by ensuring integrity, traceability, and non-repudiation of authorization-related events. Implemented on Hyperledger Besu using the QBFT consensus mechanism, this layer provides deterministic finality, fault tolerance, and strong resistance to tampering, enabling authorization decisions to be recorded as immutable and verifiable evidence (35). The blockchain layer is intentionally scoped to support governance and auditability functions rather than high-frequency clinical data processing.

By recording authorization outcomes on-chain, WiraChain extends conventional role-based access control (RBAC) mechanisms with an immutable and independently verifiable audit layer. Unlike centralized RBAC systems, where access decisions are enforced and logged within a single institutional boundary, the proposed approach enables cross-institutional verification of permission grants and revocations. This design strengthens accountability, provides non-repudiation, and reduces reliance on a single trusted authority, thereby improving security in distributed interoperability scenarios without exposing sensitive clinical data on-chain.

At the core of the on-chain layer, WiraChain records authorization outcomes through dedicated smart contracts that emit audit events indicating whether access to specific clinical resources has been granted, revoked, or overridden. These events do not store personal or clinical data. Instead, only minimal audit metadata is recorded, including pseudonymized identifiers, resource categories, action types, and timestamps. This design ensures that the blockchain functions exclusively as an immutable audit trail for authorization decisions rather than as a repository of protected health information.

From a security engineering perspective, the current implementation prioritizes architectural security properties over exhaustive adversarial evaluation. Formal threat modeling, penetration testing, and third-party smart contract security audits were not conducted as part of this study, as the primary objective was to validate architectural feasibility and interoperability rather than production readiness. Nevertheless, the attack surface of the blockchain layer is intentionally minimized: smart contracts do not store sensitive data, do not perform external contract calls, and expose a limited set of deterministic functions focused solely on permission logging. These design choices reduce common vulnerability vectors and establish a secure baseline suitable for further hardening in future deployments.

Smart contracts are developed in Solidity and deployed using Node.js, TypeScript, and the ethers.js library, following security-by-design and modularity principles. On-chain operations are limited to event emission, optimizing efficiency and minimizing resource consumption. The QBFT consensus mechanism enables low-latency block finalization, while secure key management across validator nodes and indexed blockchain events support reliable correlation between on-chain audit evidence and off-chain clinical records.

Together, these components establish a scalable, interoperable, and auditable infrastructure aligned with healthcare information security requirements. In addition to standard authorization auditing, WiraChain supports exceptional clinical scenarios through a controlled emergency (“break-glass”) access mechanism.

Emergency access can be activated only by predefined clinical roles under exceptional circumstances, such as life-threatening situations where prior patient consent cannot be obtained. Each activation is explicitly marked as an emergency event and immutably recorded on-chain, including the actor role, timestamp, and scope of access. These events are subject to post-incident review by institutional oversight entities, enabling misuse detection and accountability enforcement. Patient notification and organizational response procedures are handled off-chain in accordance with institutional policies and applicable regulations. While the break-glass mechanism does not aim to prevent misuse entirely, it ensures that any emergency access is transparent, traceable, and auditable.

In addition to standard authorization auditing, WiraChain supports exceptional clinical scenarios through a controlled emergency (“break-glass”) access mechanism. Emergency access can be activated only by predefined clinical roles under exceptional circumstances, such as life-threatening situations where prior patient consent cannot be obtained. Each break-glass activation is explicitly marked as an emergency event and immutably recorded on-chain, including the actor role, timestamp, and scope of access. These events are subject to post-incident review by institutional oversight entities, enabling detection of misuse and enforcement of accountability measures. Patient notification procedures and organizational responses are handled off-chain in accordance with institutional policies and applicable regulations. While the break-glass mechanism does not prevent misuse by itself, it ensures that any emergency access is transparent, traceable, and auditable.

### Interoperability layer

This component, developed using the HAPI FHIR framework, implements a RESTful API compliant with the HL7 FHIR standard to enable the creation, validation, and exchange of interoperable clinical resources. It functions as the central integration point for data orchestration across heterogeneous healthcare systems, ensuring semantic consistency, traceability, and data integrity in distributed environments. HAPI FHIR was selected for its maturity, full compliance with FHIR specifications, and seamless integration with Java, facilitating adaptability to local healthcare contexts in alignment with HL7 FHIR R4 and ISO/IEC 27001 information security principles (36).

The implementation adheres to the Peruvian Ministry of Health's interoperability framework established by Ministerial Resolution No. 080-2022/MINSA (37), as well as to Law No. 29733 on personal data protection (38). With respect to regulatory compliance, WiraChain is designed to align with the principles established by this legal framework, as well as internationally recognized data protection regulations such as HIPAA and GDPR. This alignment is achieved through data minimization, off-chain storage of clinical information, patient-controlled authorization mechanisms, and immutable audit logging.

It is important to note that no formal regulatory compliance certification or legal accreditation was pursued within the scope of this research. Such certification processes require institutional deployment, organizational controls, and regulatory audits that exceed the objectives of an academic prototype. Consequently, regulatory compliance is treated as a design objective rather than a certified guarantee.

The persistence architecture employs a dual storage model, combining a structured internal database for operational efficiency with a PostgreSQL database normalized according to FHIR resources, ensuring interoperability and seamless data exchange with external systems.

## Validation

This section provides a comprehensive overview of the activities undertaken to validate the proposed framework. The validation process includes simulating a representative case study and assessing the related performance metrics.

### Use case

To illustrate the interaction between the application and various patients, as well as the mechanisms for information exchange, a representative use case was developed based on the approach outlined by Mandarino et al. (39). This case study implements an interoperability solution for a healthcare clinic in Lima, Peru, using the HL7 FHIR standard to integrate diverse clinical systems with a private blockchain network that serves as a distributed ledger. This configuration provides a structured methodology for demonstrating the technical feasibility and data exchange protocols of the proposed model, creating a foundation for subsequent clinical validation. While the simulation is a necessary first step in a controlled environment, we acknowledge that future work must involve pilot deployments in operational healthcare settings to fully assess workflow integration, usability, and regulatory compliance.

To demonstrate the platform's technical functionality, we present a simulated scenario involving Dr. Paul Jones from the Jesús Esperanza Clinic. In this scenario, Dr. Jones must provide emergency medical attention to Nathan Jack, a foreign patient without a local clinical history. Clinical data relevant to the encounter may originate from different sources, including locally registered records or manually entered information when prior digital data are unavailable. These data are incorporated into the platform through institutional clinical workflows and managed according to WiraChain's internal data model. Interoperability mechanisms operate independently of the underlying distributed ledger, allowing data originating from paper-based records or non-DLT interoperability systems to be incorporated without requiring blockchain support at the data source. This design was validated to demonstrate a core architectural principle: when information exchange with external entities is required, selected data are exposed through standardized HL7 FHIR resources. The process begins with a federated query executed through the FHIR API, which retrieves standardized clinical resources from an external server. Each stage of the interaction is recorded immutably on the blockchain network through transactions containing cryptographic hash values, enabling traceability and integrity verification without storing clinical content on-chain. This simulation serves as a proof of concept for the technical workflow, which is a prerequisite for engaging in real-world clinical trials.

To support interoperability among systems at the Jesús Esperanza Clinic using WiraChain, several preparatory steps were undertaken. The platform was deployed to define its purpose, functionalities, and role in interoperability and auditability. Clinical records were registered within the platform's internal data model and, when necessary, mapped to HL7 FHIR representations to enable standardized exchange across institutional boundaries. Authorized users, including patients, clinicians, and administrators, were registered, and training sessions were conducted to familiarize staff with the platform, the HL7 FHIR standard, and blockchain-based auditing mechanisms. These sessions were instrumental in refining the system's interface and procedures; however, we recognize that formal, empirical usability studies and clinical user acceptance testing with healthcare professionals in live environments constitute essential future work to address practical adoption barriers.

For the simulation, the complete application stack was containerized and deployed within an isolated AWS instance. All components operated in a controlled environment to ensure reproducibility and consistency of the initial technical evaluation. The main steps of this testing procedure are described below. This synthetic validation establishes a necessary baseline for functionality, security, and data integrity prior to the significant logistical and regulatory complexities of a clinical pilot study.

#### Patient authorization

**Figure 4:**
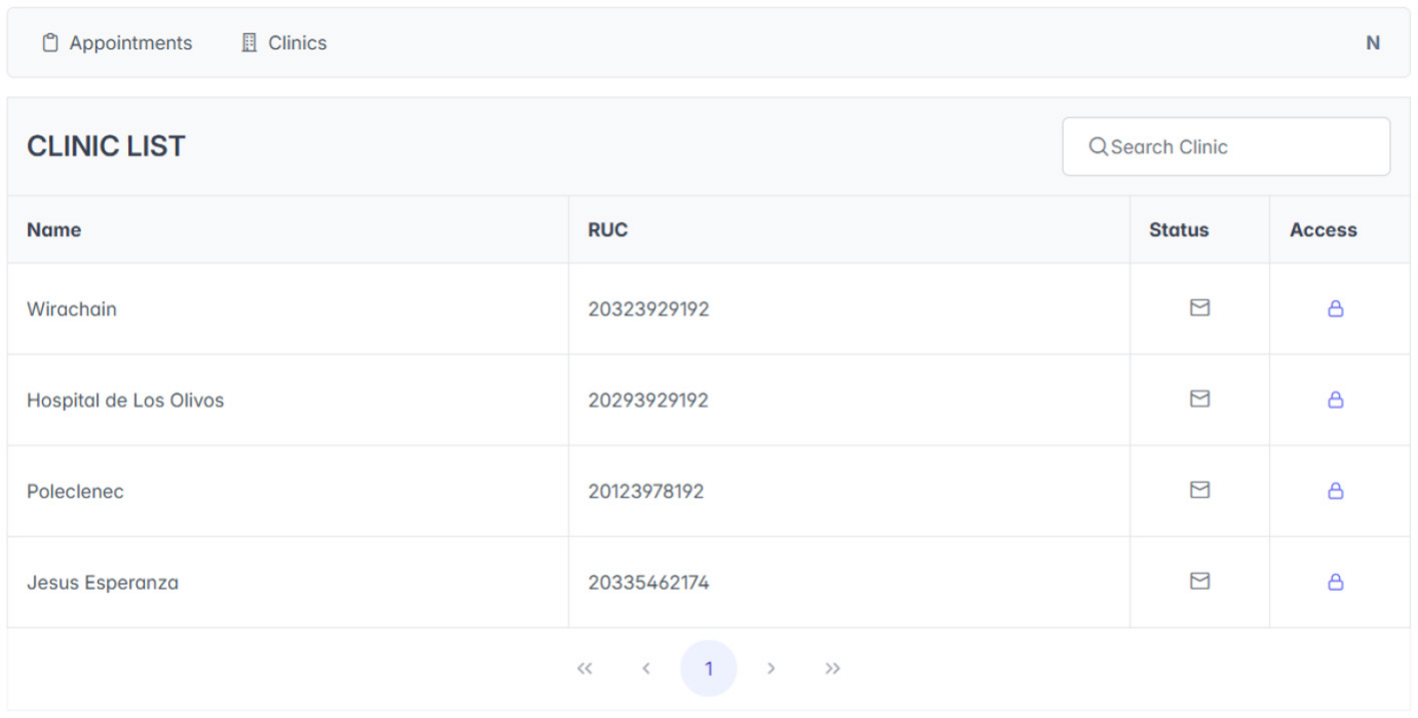
Patient authorization.

As illustrated in Figure 4, the patient must give explicit consent to the clinic they will visit. This consent allows authorized personnel to create and manage the patient's clinical records within the system. This process ensures that access to and management of data comply with established authorization policies and patient privacy regulations.

#### Medical record visualization

**Figure 5:**
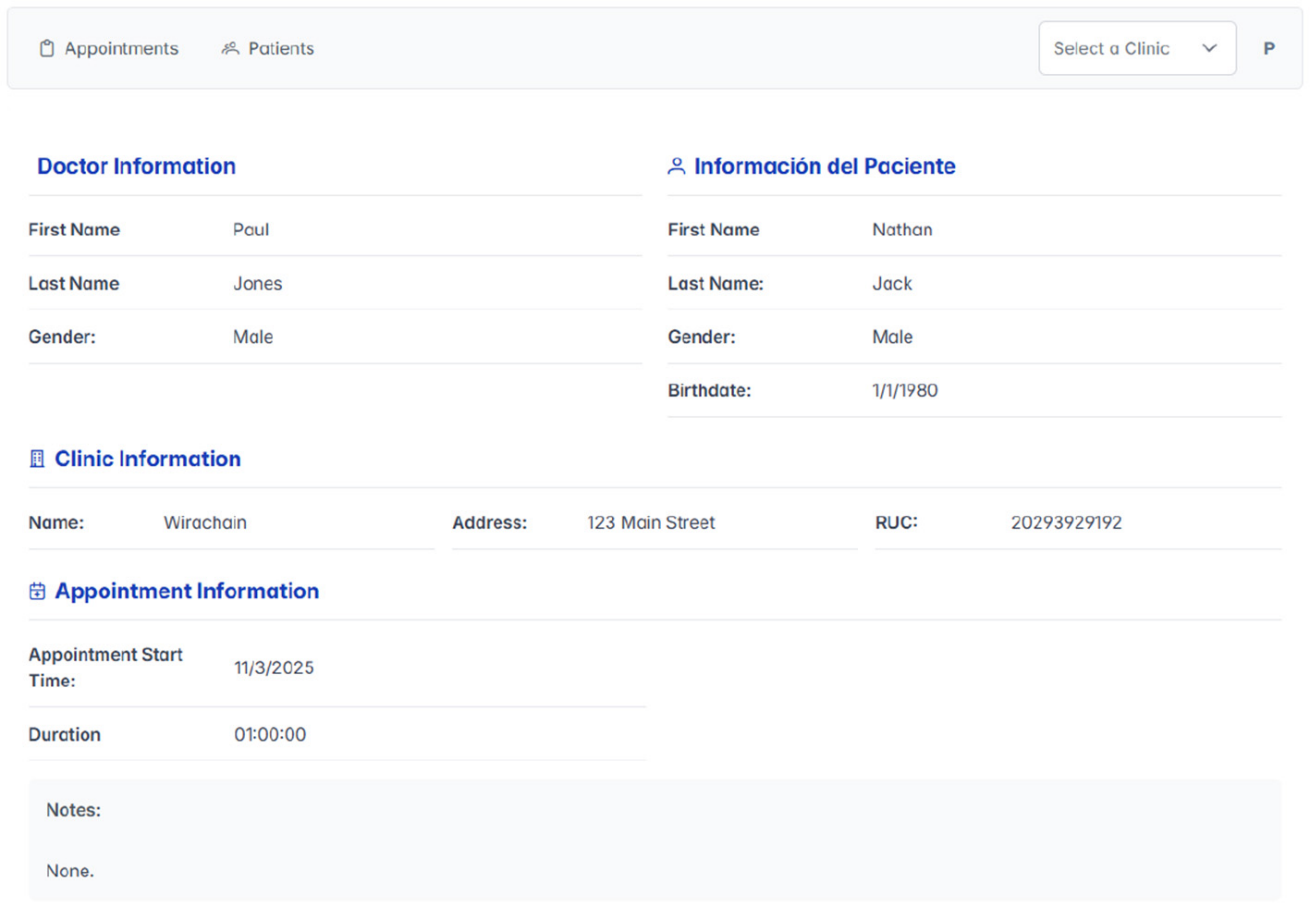
Medical record visualization.

Once the patient gives the necessary permissions for record creation, the attending doctor is granted access to the patient's clinical history. This access is crucial for ensuring continuity of care and supporting informed medical decisions, as shown in Figure 5.

#### Creation of clinical consultation

**Figure 6:**
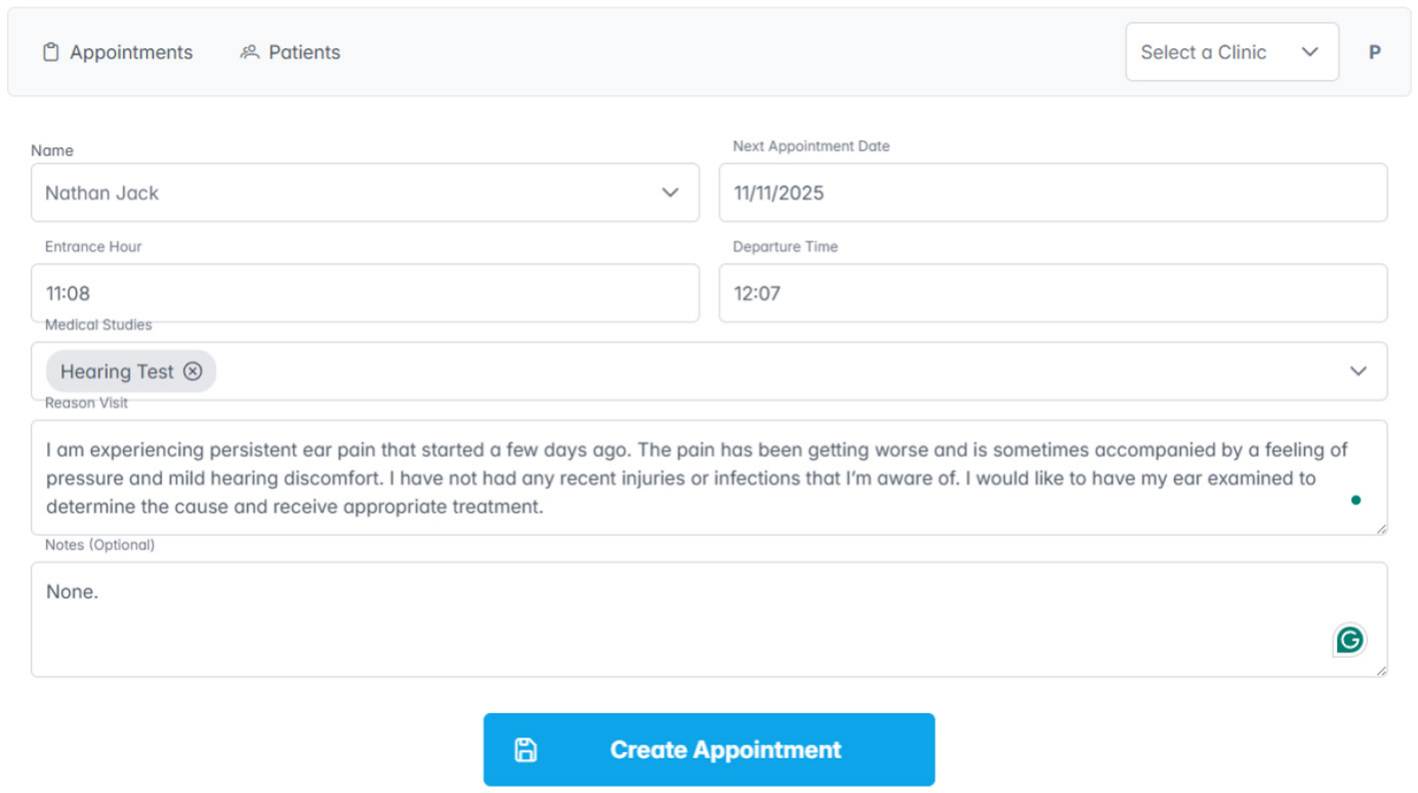
Creation of clinical consultation.

During the medical consultation, the doctor is authorized to create a new clinical record directly linked to the patient. This record systematically documents all relevant information required for monitoring the patient's health status, as shown in Figure 6.

#### Importing patient data

**Figure 7:**
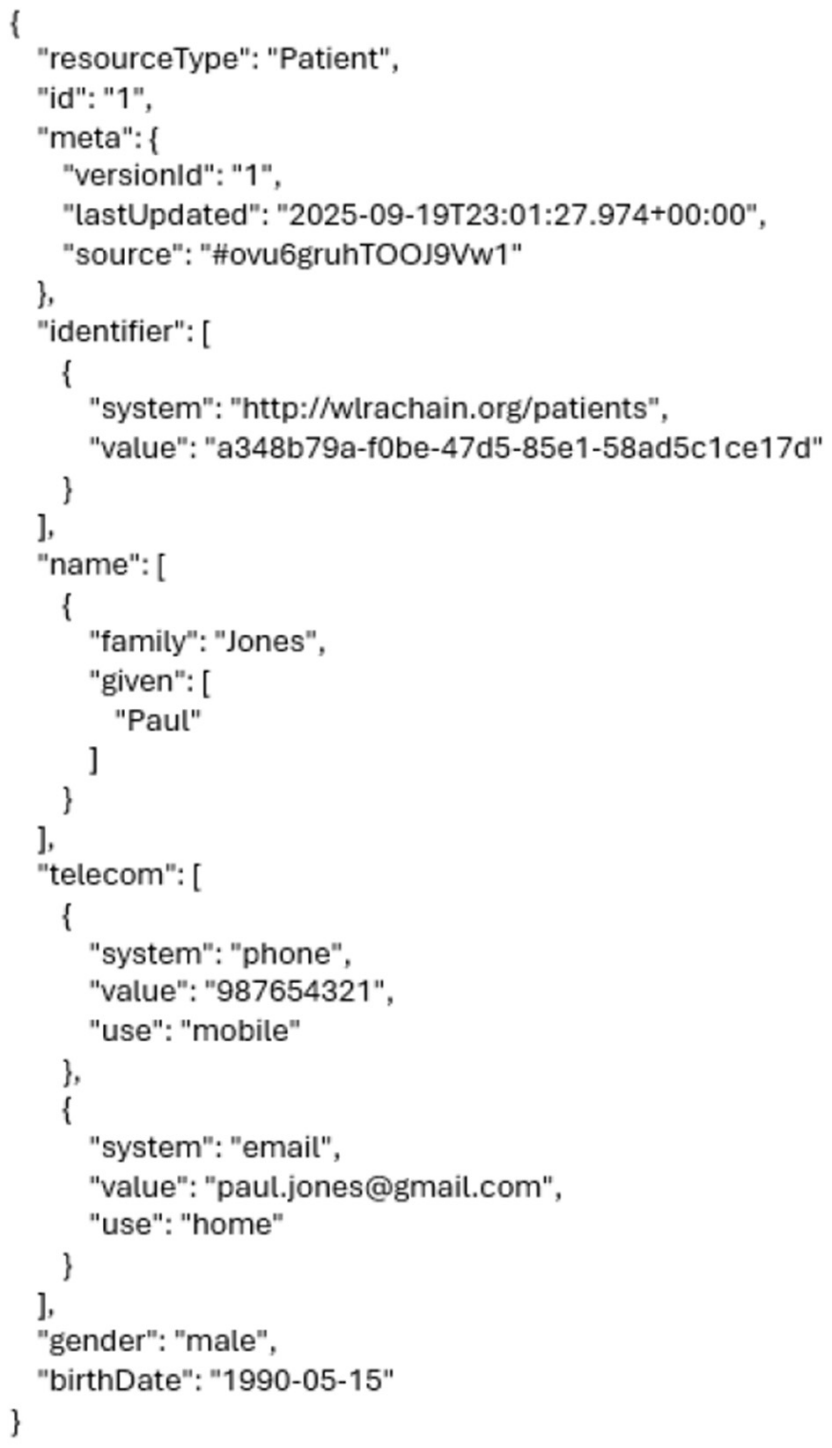
Importing patient data.

Once the doctor completes the patient's consultation, the details are securely registered on the blockchain network. This immutable record can later be accessed by other authorized clinics, enhancing data portability and interoperability across different institutions, as shown in Figure 7.

For the off-chain and interoperability layer, a t3.large instance was employed, which features two vCPUs and 8 GiB of RAM, costing approximately US $0.0832 per hour (≈ US $59.90 per month). On the other hand, the on-chain layer, responsible for running the Hyperledger Besu nodes and the QBFT consensus, used a t3.2xlarge instance. This instance offers eight vCPUs and 32 GiB of memory, with an estimated cost of US $0.3328 per hour (≈ US $239.60 per month) (40).

The T3 family of instances provides a balanced combination of compute power, memory, and network performance. These instances feature burstable CPU capabilities that allow for temporary performance boosts by utilizing accumulated CPU credits. This configuration is particularly well-suited for workloads with variable utilization patterns, such as interoperability services, blockchain node operations, and lightweight off-chain data management.

### Metrics

The performance evaluation of the Wirachain platform was conducted by analyzing two key metrics: (1) transactions per second and (2) latency. To achieve this, load tests were performed to simulate the operating conditions of a production environment. The aim was to replicate the actual behavior of the system under varying levels of demand in a controlled manner.

These tests are crucial for measuring the platform's processing capacity, identifying potential bottlenecks, and validating its scalability and stability before large-scale deployment. This process ensures optimal performance in real-world operating conditions (24). The experimental setup included a Hyperledger Besu blockchain network utilizing the QBFT consensus mechanism, which was deployed on an Amazon EC2 t3.2xlarge cloud computing instance. To conduct the load tests, a performance script was created using the ethers.js v6 library.

Scripts were designed to measure throughput (Transactions Per Second - TPS) and latency by executing a large number of concurrent transactions that triggered the critical functions of Wirachain smart contracts deployed on the network. This method enables us to obtain specific and relevant performance metrics that reflect the platform's actual operations. Following the methodology proposed by Sharma et al. (41), this work incorporates key metrics for performance analysis: Throughput (TPS) and Average Latency (ms). Throughput indicates the number of transactions completed per second during a given time interval and is calculated using the following formula:


$$\begin{array}{l} \text{TPS}=\frac{N}{T} \end{array}$$ (1)

where *N* is the total number of transactions processed and *T* denotes the total execution time of the experimental batch (in seconds).


$$\begin{array}{l} \text{Average Latency}=\frac{1}{N}\overset{N}{\underset{i=1}{\sum }}L_{i} \end{array}$$ (2)

where *L*_*i*_ represents the latency measured for the *i*-th transaction and *N* is the total number of transactions in the batch.

#### Transaction per second

Tests were conducted, similar to those by Sharma et al. (42), Mythili et al. (43), and Kaushal et al. (44), to measure transactions per second. These tests focused on the Grant and Revoke operations of the smart contract developed under the QBFT consensus protocol, aiming to evaluate its performance and scalability. The testing utilized Ethers.js for issuing transactions in parallel and Solidity for the contract logic. Two environments were considered for these tests: (i) a local environment equipped with eight physical cores (16 threads) and 32 GB of RAM, and (ii) an AWS t3.2xlarge instance, which represents a distributed validation scenario.

**Figure 8:**
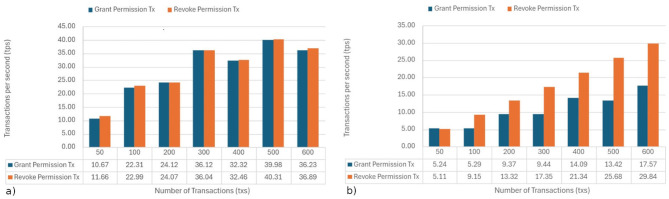
Transactions per second: **(a)** local instance; **(b)** AWS t3.2xlarge instance.

Each experiment involved simultaneously sending between 50 and 600 transactions while recording the performance in transactions per second (TPS). In the local environment (see Figure 8a), we achieved rates of approximately 40 TPS for both Grant and Revoke, indicating good parallel processing capability and low internal latency. However, in the AWS instance (see Figure 8b), the maximum rates were around 30 TPS for Revoke and 17 TPS for Grant. The lower performance in this environment can be attributed to network latency and resource virtualization limitations.

The system demonstrated a consistent and stable response, confirming that its performance is suitable for web or distributed deployment. The differences observed between environments are expected, as the local machine has a superior configuration with 16 threads and 32 GB of RAM, whereas the AWS instance operates in a virtualized environment with less computing power. Overall, the results validate the efficiency and feasibility of the proposed model and confirm the effective adaptation of the QBFT consensus for parallel and transaction-intensive execution scenarios.

#### Latency

According to the proposals by Hashim et al. (45), Ravi et al. (46), and Alhusayni et al. (47), latency was assessed to evaluate the system's responsiveness as transaction volume increased progressively. The tests were conducted in two environments identical to those defined for the TPS benchmark: one local and one in the cloud, both featuring equivalent architecture. Consistent load and configuration conditions were maintained throughout the tests.

**Figure 9:**
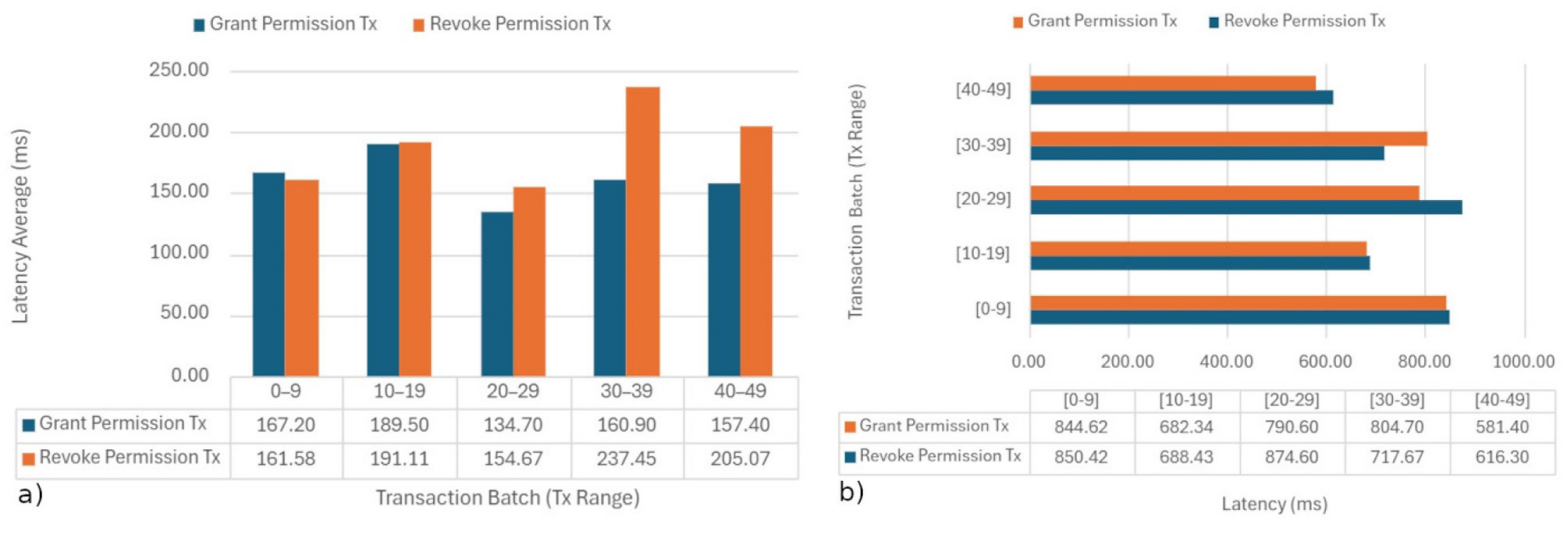
Transaction latency: **(a)** local instance; **(b)** AWS t3.2xlarge instance.

The latency evaluation indicates that permission-related blockchain operations exhibit stable and predictable behavior under batch-based transaction execution. As shown in Figures 9a, b, the local environment maintained average latencies between 134 and 237 ms, while the cloud deployment presented higher values ranging from 580 to 900 milliseconds, primarily due to virtualization and network overhead. Transactions were intentionally executed in batches to emulate realistic access patterns, where authorization and revocation requests occur in grouped administrative or clinical events rather than as isolated high-frequency actions. This batching approach reduces consensus overhead, improves throughput efficiency, and provides a more accurate representation of operational workloads in healthcare settings.

Despite higher latency in the cloud environment, the observed performance remains within the expected range for Hyperledger Besu networks and should be interpreted within the specific scope of WiraChain's blockchain layer. The system is not designed to process high-frequency clinical transactions on-chain; instead, blockchain interactions are limited to sporadic permission grant, revocation, and audit events, executed in batches. Time-sensitive clinical data access and exchange occur off-chain through optimized HL7 FHIR services, ensuring that diagnostic and treatment workflows are not constrained by blockchain throughput or latency.

A latency of under 1,000 ms for a permission update operation is considered excellent from a usability standpoint. When a physician or patient interacts with the Wirachain interface, they would perceive the confirmation of their actions as nearly instantaneous. This rapid response is critical for adoption in clinical environments, where quick interactions are essential. Thus, the decentralized permission system ensures that no noticeable delays are introduced.

**Figure 10:**
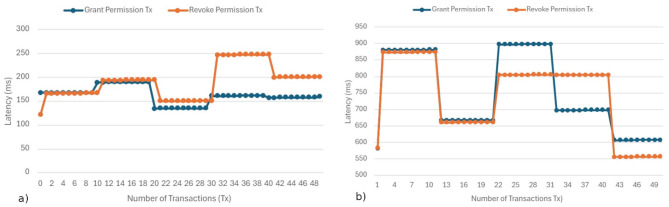
Latency profile: **(a)** local instance; **(b)** AWS t2.large instance.

Figure 10a shows the complete latency distribution recorded in the local environment. It reveals a consistent concentration between 130 and 240 ms, with no significant outliers. This pattern indicates low temporal dispersion and effective management of concurrent processing, which can be attributed to the optimized execution flow in the RPC client. The stability of these measurements confirms that the QBFT consensus response time remains reliable even with slight variations in load, ensuring system predictability in local development or validation settings.

In contrast, Figure 10b illustrates the accumulated latencies of the AWS t3.2xlarge instance, showing fluctuations between 580 and 900 ms. This indicates a sustained increase compared to the local environment. However, despite this difference, the latency behavior remains consistent and free from abrupt deviations. This suggests that the challenges posed by the virtualized environment arise primarily from external factors, such as hypervisor overhead and network latency, rather than from the contract logic or consensus mechanism itself. These findings demonstrate that although the cloud environment introduces additional delays, the temporal integrity and stability of the Besu network are maintained, confirming its suitability for distributed clinical interoperability and high-availability scenarios.

## Conclusion

After implementing the WiraChain application, which enables the management of electronic records and facilitates their exchange with international standards such as HL7 FHIR, it is concluded that this tool significantly contributes to improving interoperability within a clinical environment.

In the first validation phase, a use case was implemented with the objective of demonstrating the main workflow of the application, the essential functions of the different user types, as well as the user interface and the data it requires.

In this use case, the process of patient permission management, the creation of a medical record, and its subsequent interoperability through transformation to the FHIR format were simulated. This aimed to demonstrate that the proposed solution effectively enhances interoperability in clinical environments.

Latency evaluation showed an average transaction time of 162 ms, demonstrating that the event-driven model provides efficient execution for permission grant and revocation operations. This performance ensures agile responses without compromising security or traceability, validating the suitability of the approach for clinical scenarios where interactions occur sporadically.

In terms of performance, stress tests conducted with Node.js and Ethers.js on the Hyperledger Besu network reached a maximum throughput of 40 transactions per second (TPS), maintaining stability under increasing load. This result confirms the scalability, resilience, and practical viability of the proposed architecture for regional hospital networks, consolidating its potential as a sustainable solution for decentralized clinical interoperability and secure management of digital health consents.

Among the study's limitations is the cautious attitude of clinics regarding the exchange of information about their systems and procedures. Additionally, the study was conducted within a limited timeframe, which constrained the scope of the implementation. As a result, the application primarily focused on the management of medical appointments and related clinical interactions, without addressing complex scenarios such as surgical procedures or the handling of major medical conditions.

Another relevant line of research would be the incorporation of advanced mechanisms for access control and data anonymization, in order to strengthen patient privacy in accordance with international regulations. Finally, it is proposed to explore the application of emerging technologies, such as artificial intelligence and machine learning, to optimize the management and analysis of electronic health records.

These future directions align with broader trends in smart healthcare, where explainable AI methods are being analyzed for clinical decision support systems, addressing both technical and usability challenges (48). Similarly, comprehensive reviews of AI capabilities in smart healthcare highlight the potential for AI-enhanced interoperability platforms like WiraChain to evolve into more intelligent, adaptive systems (49).

This work contributes to strengthening the theoretical framework related to interoperability in health information systems by demonstrating the feasibility of integrating the HL7 FHIR standard with blockchain technologies for secure and decentralized management of electronic health records.

In practice, the implementation of this solution optimizes the management of electronic health records (EHRs), ensuring greater transparency, traceability, and security in the exchange of information between medical institutions. Likewise, its adoption can help reduce errors derived from data fragmentation and facilitate continuity of patient care. Collectively, these implications reinforce the usefulness of the proposed solution as a supporting tool for the digitalization and modernization of healthcare systems.

## Data Availability

The original contributions presented in the study are included in the article/supplementary material, further inquiries can be directed to the corresponding author.
